# Supplementary material for: CREsted: modeling genomic and synthetic cell-type-specific enhancers across tissues and species
Source: Nat Methods. 2026 Apr 2;23(5):946–59. doi: 10.1038/s41592-026-03057-2 (PMC13167471; doi:10.1038/s41592-026-03057-2)
Supplement: Supplementary file 1 — Supplementary Figs. 1–10, Notes 1–5 and References 102–135. [file 41592_2026_3057_MOESM1_ESM.pdf]

nature methods

Supplementary information https://doi.org/10.1038/s41592-026-03057-2

CREsted: modeling genomic and synthetic cell-type-specific enhancers across tissues and species

In the format provided by the authors and unedited

CREsted: modeling genomic and synthetic cell type-specific enhancers across tissues and species

Supplementary Information

This file contains:

- Supplementary Figures S1-10
- Supplementary Notes S1-5
- Supplementary References 102-135

Supplementary Figures

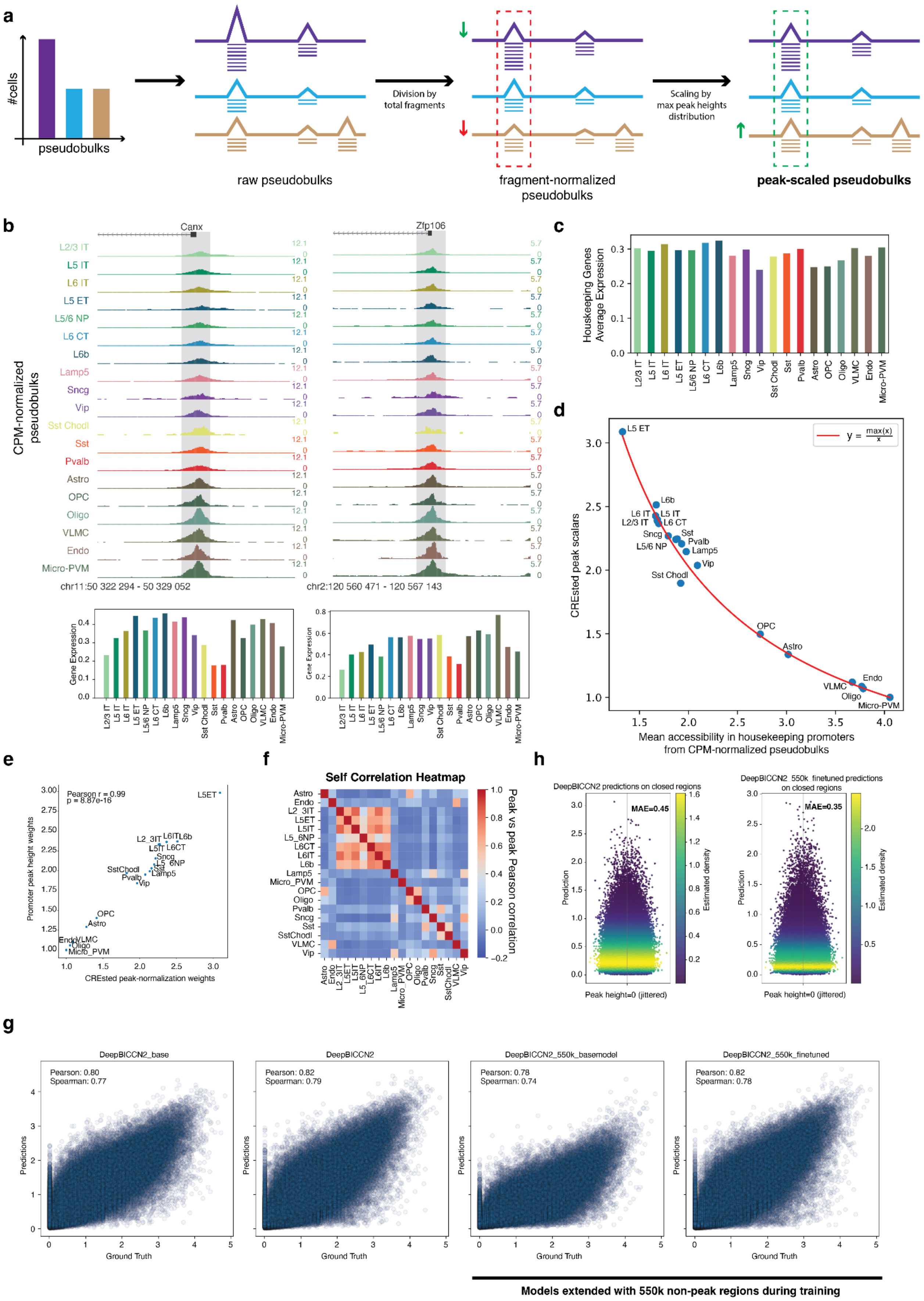

a
#cells
pseudobulks
raw pseudobulks
Division by
total fragments
fragment-normalized
pseudobulks
Scaling by
max peak heights
distribution
peak-scaled pseudobulks
b
Canx
Zfp106
CPM-normalized
pseudobulks
L2/3 IT
L5 IT
L6 IT
L5 ET
L5/6 NP
L6 CT
L6b
Lamp5
Sncg
Vip
Sst Chodl
Sst
Pvalb
Astro
OPC
Oligo
VLMC
Endo
Micro-PVM
chr11:50 322 294 - 50 329 052
chr2:120 560 471 - 120 567 143
Gene Expression
c
Housekeeping Genes
Average Expression
d
CREsted peak scalars
Mean accessibility in housekeeping promoters
from CPM-normalized pseudobulks
e
Pearson r = 0.99
p = 8.87e-16
Promoter peak height weights
CREsted peak-normalization weights
f
Self Correlation Heatmap
Peak vs peak Pearson correlation
h
DeepBICCN2 predictions on closed regions
MAE=0.45
DeepBICCN2_550k_finetuned predictions
on closed regions
MAE=0.35
Prediction
Estimated density
Peak height=0 (jittered)
g
DeepBICCN2_base
Pearson: 0.80
Spearman: 0.77
DeepBICCN2
Pearson: 0.82
Spearman: 0.79
DeepBICCN2_550k_basemodel
Pearson: 0.78
Spearman: 0.74
DeepBICCN2_550k_finetuned
Pearson: 0.82
Spearman: 0.78
Predictions
Ground Truth
Models extended with 550k non-peak regions during training

Figure S1. Overview of peak scaling method and non-peak extension during model training.

(**a**) Overview of the peak normalization method. (**b**) Peak heights over all cell types of the housekeeping *Canx* and *Zfp106* genes from standard CPM-normalized scATAC-seq tracks (top). Normalized expression of both genes over all cell types (bottom). (**c**) Average expression for all housekeeping genes (n=3,969 genes) over all cell types. (**d**) Comparison of average peak heights in housekeeping promoters and the peak scalars used to counterbalance them. The expected curve is shown in red. (**e**) Comparison of CREsted peak-normalization weights and the weights obtained from looking at housekeeping promoter regions. (**f**) Heatmap indicating Pearson correlation of log-transformed peak heights across cell types from a set of cell type-specific regions. (**g**) Scatter plot of predictions vs peak heights over all classes for the DeepBICCN2 base model, DeepBICCN2 model, Non-peak extended base model, and Non-peak extended fine-tuned model. Regions are held-out test set regions from the filtered cell type-specific test set (n=8,198 regions). (**h**) Closed region predictions over all classes on closed regions (n=157,998 regions) from the different models. MAE: mean absolute error.

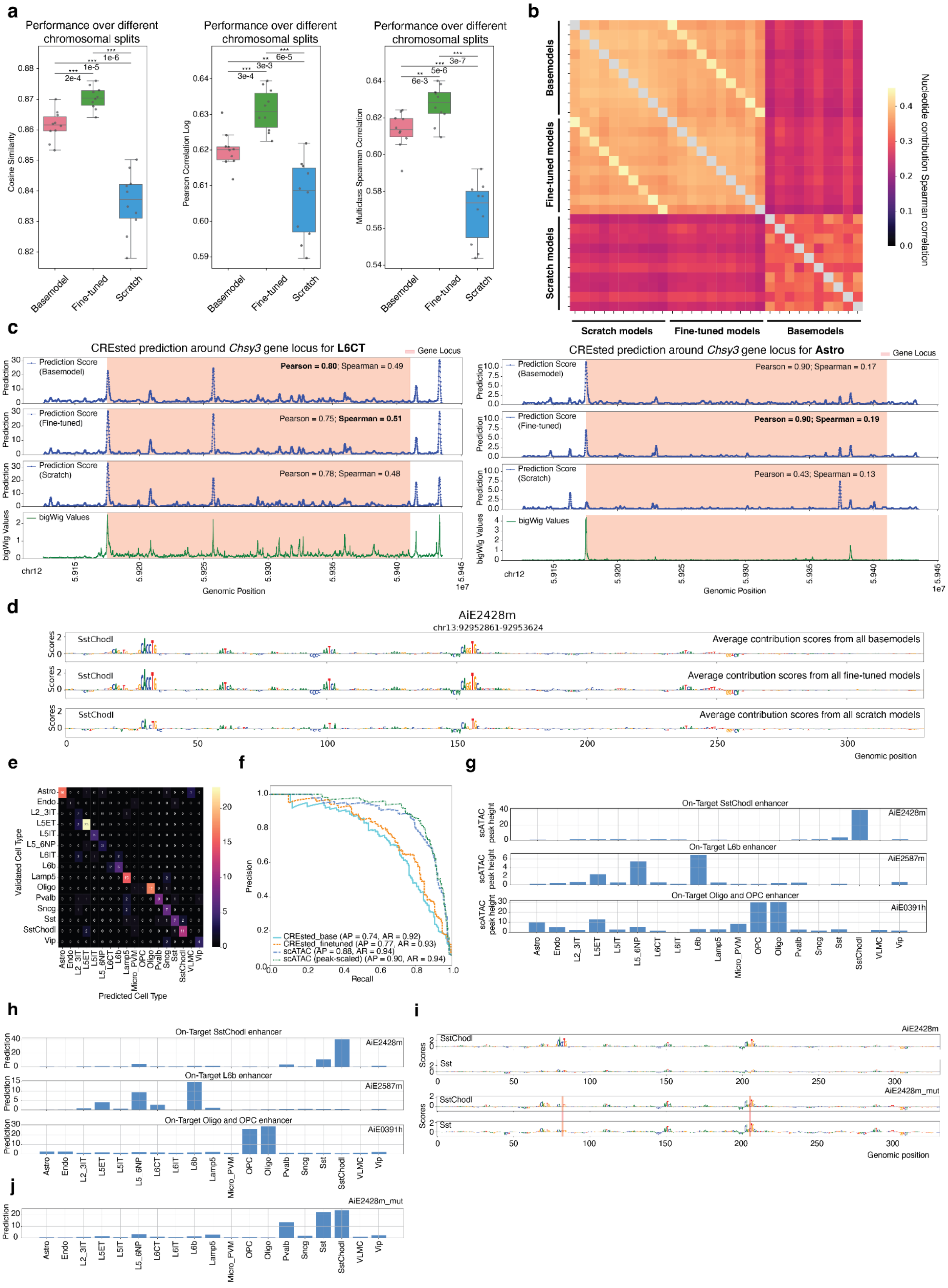
a
Performance over different chromosomal splits
Cosine Similarity
Pearson Correlation Log
Multiclass Spearman Correlation
Basemodel
Fine-tuned
Scratch
2e-4
1e-5
1e-6
3e-4
3e-3
6e-5
6e-3
5e-6
3e-7
b
Basemodels
Fine-tuned models
Scratch models
Nucleotide contribution Spearman correlation
c
CREsted prediction around Chsy3 gene locus for L6CT
Gene Locus
Prediction Score (Basemodel)
Pearson = 0.80; Spearman = 0.49
Prediction Score (Fine-tuned)
Pearson = 0.75; Spearman = 0.51
Prediction Score (Scratch)
Pearson = 0.78; Spearman = 0.48
bigWig Values
Genomic Position
CREsted prediction around Chsy3 gene locus for Astro
Pearson = 0.90; Spearman = 0.17
Pearson = 0.90; Spearman = 0.19
Pearson = 0.43; Spearman = 0.13
d
AiE2428m
chr13:92952861-92953624
SstChodl
Average contribution scores from all basemodels
Average contribution scores from all fine-tuned models
Average contribution scores from all scratch models
Scores
Genomic position
e
Validated Cell Type
Predicted Cell Type
Astro
Endo
L2_3IT
L5ET
L5IT
L5_6NP
L6CT
L6IT
L6b
Lamp5
Micro_PVM
OPC
Oligo
Pvalb
Sncg
Sst
SstChodl
VLMC
Vip
f
Precision
Recall
CREsted_base (AP = 0.74, AR = 0.92)
CREsted_finetuned (AP = 0.77, AR = 0.93)
scATAC (AP = 0.88, AR = 0.94)
scATAC (peak-scaled) (AP = 0.90, AR = 0.94)
g
On-Target SstChodl enhancer
AiE2428m
On-Target L6b enhancer
AiE2587m
On-Target Oligo and OPC enhancer
AiE0391h
scATAC peak height
h
Prediction
i
AiE2428m
SstChodl
Sst
AiE2428m_mut
Scores
Genomic position
j
AiE2428m_mut

Figure S2. Comparison of base-, fine-tuned, and scratch models over different chromosomal splits and CREsted on *in vivo* validated mouse cortex enhancers.

(**a**) Comparison of model performances from the 10 differentially chromosomally split base-models, fine-tuned models and scratch models. Each dot in the figure represents the average value of the relevant metric across these 19 cell types. Pairwise differences between models were assessed using two-sided Welch's t-tests on the metrics, and the resulting P values were adjusted for multiple comparisons using the Benjamini–Hochberg false discovery rate procedure; exact adjusted P values for each model–model comparison are reported in the figure. * < 0.05, ** < 0.01, *** < 0.001. (**b**) Heatmap of nucleotide contribution score Spearman correlations calculated a set of 171 in vivo validated enhancers[17] for their target class. A set of 10 chromosomal splits were used per model type. (**c**) Gene locus prediction plots of the *Chsy3* locus, using the average predictions from all differentially chromosomally split base-models, fine-tuned models, and scratch models, for L6CT (left) and Astrocytes (Astro) (right). Pearson and Spearman correlations between the predicted accessibility tracks of the Chsy3 gene locus from the average predictions of the basemodels, fine-tuned models and scratch models for L6CT and Astro are indicated in the plots. (**d**) Contribution score comparison for example region with different model training strategies. Contribution scores were the average over all chromosomal splits per strategy. Region coordinates: chr13:92952861-92953624 (mm10). (**e**) Heatmap of predicted labels from the maximum prediction per region and the targeted validated cell types for a set of 171 *in vivo* validated enhancers. (**f**) Averaged multi-label precision-recall curve for predicting the set of validated enhancers indicating specificity per cell types. Specificity was calculated for scATAC peaks (scaled and non-scaled), and for CREsted predictions (basemodel and fine-tuned). The scATAC curves represent how well solely using accessibility values over the cell types predicts enhancer activity. (**g**) scATAC peak heights for three example enhancer regions. (**h**) Predictions for three example enhancer regions. (**i**) Contribution scores for the AiE2428m SstChodl enhancer, before (top) and after (bottom) E-box motif mutation. (**j**) Prediction scores for mutated AiE2428m enhancer. For boxplots in a the top/lower hinge represents the upper/lower quartile and whiskers extend from the hinge to the largest/smallest value no further than 1.5 × interquartile range from the hinge, respectively. The median is used as the center.

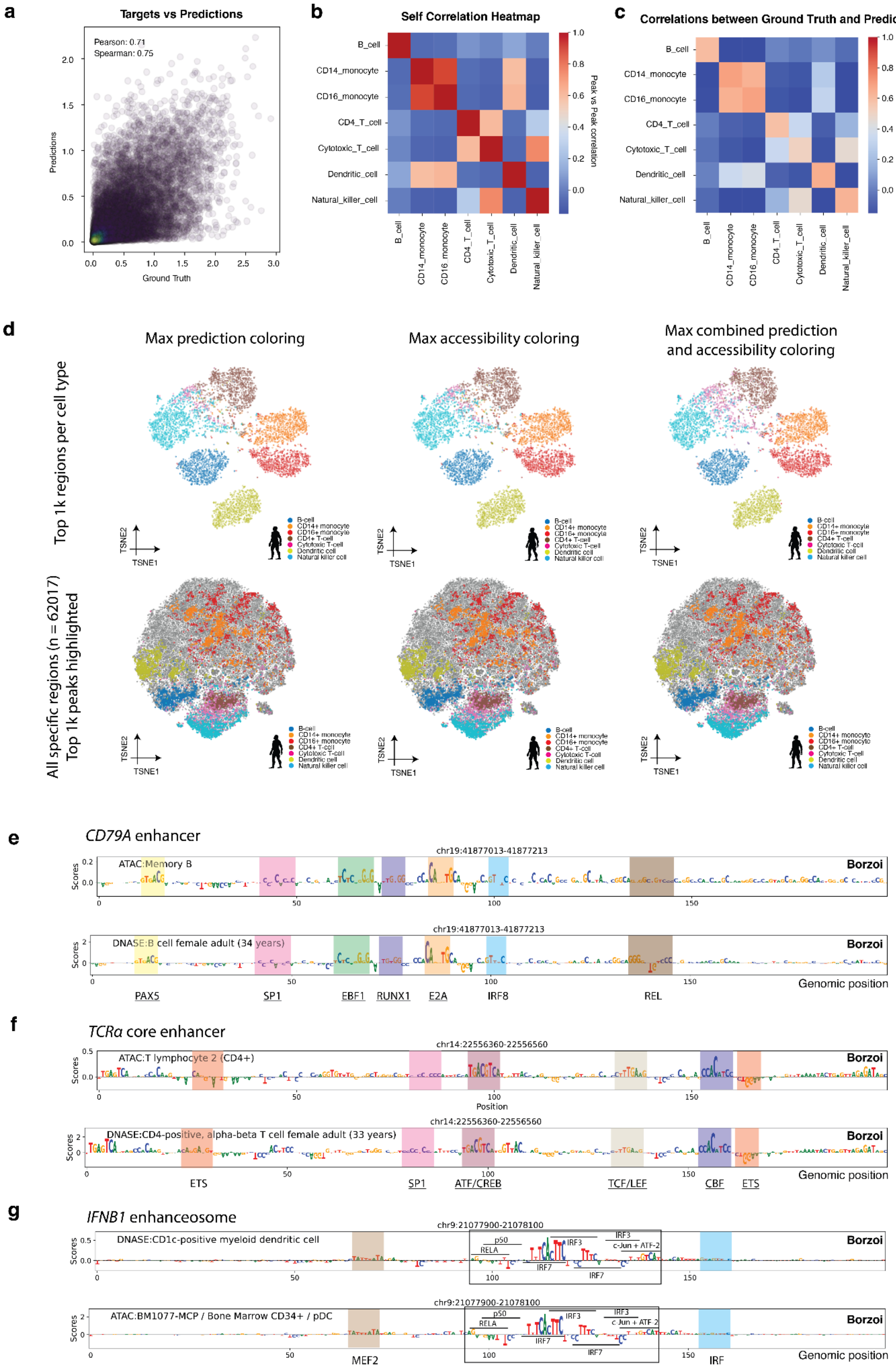

a
Targets vs Predictions
Pearson: 0.71
Spearman: 0.75
Predictions
Ground Truth
b
Self Correlation Heatmap
B_cell
CD14_monocyte
CD16_monocyte
CD4_T_cell
Cytotoxic_T_cell
Dendritic_cell
Natural_killer_cell
Peak vs Peak correlation
c
Correlations between Ground Truth and Predictions
Prediction vs Peak correlation
d
Max prediction coloring
Max accessibility coloring
Max combined prediction and accessibility coloring
Top 1k regions per cell type
All specific regions (n = 62017)
Top 1k peaks highlighted
TSNE1
TSNE2
B-cell
CD14+ monocyte
CD16+ monocyte
CD4+ T-cell
Cytotoxic T-cell
Dendritic cell
Natural killer cell
e
CD79A enhancer
chr19:41877013-41877213
ATAC:Memory B
Borzoi
Scores
DNASE:B cell female adult (34 years)
PAX5
SP1
EBF1
RUNX1
E2A
IRF8
REL
Genomic position
f
TCRα core enhancer
chr14:22556360-22556560
ATAC:T lymphocyte 2 (CD4+)
Position
DNASE:CD4-positive, alpha-beta T cell female adult (33 years)
ETS
SP1
ATF/CREB
TCF/LEF
CBF
ETS
Genomic position
g
IFNB1 enhanceosome
chr9:21077900-21078100
DNASE:CD1c-positive myeloid dendritic cell
p50
RELA
IRF3
IRF7
c-Jun + ATF-2
ATAC:BM1077-MCP / Bone Marrow CD34+ / pDC
MEF2
IRF
Genomic position

Figure S3. Fine-tuned CREsted PBMC model performance overview, embeddings, and Borzoi comparison.

(**a**) Scatter plot of all log-transformed predicted targets and predictions for all specific test set regions (n=4,303 regions) over all classes. (**b**) Accessibility correlation of log-transformed specific test region (n=4,303 regions) peak heights across cell types. (**c**) Correlation of log-transformed predictions and accessibility over cell types for specific test set regions (n=4,303 regions). (**d**) Embeddings of the second-to-last DeepPBMC model layer of the top 1,000 regions per cell type (top), and of all cell type-specific peaks with only the top 1,000 regions per cell type highlighted (bottom). The left column colors regions by their max DeepPBMC prediction value, the middle column by their max peak height, and the right column by their average. (**e**) Contribution scores from the base Borzoi model for the *CD79A* (hg38 chr19:41,876,056-41,878,170) and *TCRα* core (hg38 chr14:22,555,403-22,557,517) (**f**) enhancers and the *IFNB1* enhanceosome (hg38 chr9:21,076,963-21,079,077) (**g**) for a selection of relevant classes. Identified motifs are highlighted and annotated. Validated TFBS are underlined.

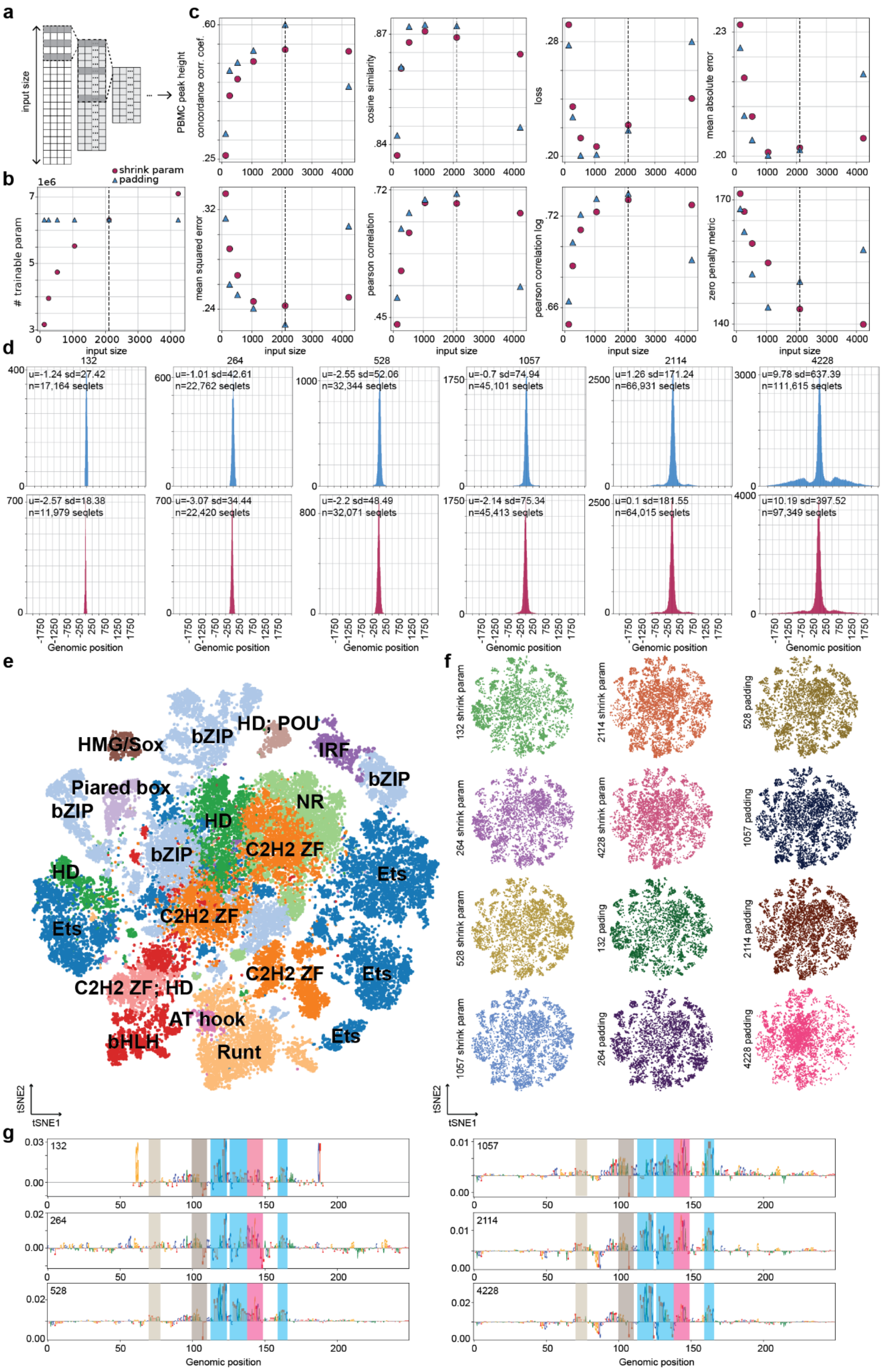
a
b
c
d
e
f
g
input size
shrink param
padding
1e6
trainable param
PBMC peak height concordance corr. coef.
cosine similarity
loss
mean absolute error
mean squared error
pearson correlation
pearson correlation log
zero penalty metric
132
264
528
1057
2114
4228
u=-1.24 sd=27.42 n=17,164 seqlets
u=-1.01 sd=42.61 n=22,762 seqlets
u=-2.55 sd=52.06 n=32,344 seqlets
u=-0.7 sd=74.94 n=45,101 seqlets
u=1.26 sd=171.24 n=66,931 seqlets
u=9.78 sd=637.39 n=111,615 seqlets
u=-2.57 sd=18.38 n=11,979 seqlets
u=-3.07 sd=34.44 n=22,420 seqlets
u=-2.2 sd=48.49 n=32,071 seqlets
u=-2.14 sd=75.34 n=45,413 seqlets
u=0.1 sd=181.55 n=64,015 seqlets
u=10.19 sd=397.52 n=97,349 seqlets
Genomic position
HMG/Sox
bZIP
HD; POU
IRF
Piared box
NR
HD
C2H2 ZF
Ets
C2H2 ZF; HD
AT hook
bHLH
Runt
132 shrink param
264 shrink param
528 shrink param
1057 shrink param
2114 shrink param
4228 shrink param
132 pading
264 padding
528 padding
1057 padding
2114 padding
4228 padding
tSNE1
tSNE2

Figure S4. Model input size benchmark on PBMC dataset.

(**a**) Schematic representation of a dilated convolutional neural network. (**b**). Number of trainable parameters versus input size with and without applying padding. (**c**) Performance metrics versus input size with and without applying padding. (**d**) Distribution of seqlet locations versus input size without (top) and with (bottom) padding. (**e**) tSNE of 6,000 seqlets based on seqlet similarities colored based on TF-family calculated using TF-MInDi. (**f**) tSNE as in (e) split by input size and whether padding was used (padding) or not (param. shrink). (**g**) Contribution scores of the IFNB1 enhanceosome (hg38 chr9:21,076,963-21,079,077, zoomed to center 250 bp) for the dendritic cell class for models with different input sizes without using padding. Seqlets are annotated using colors according to panel e. Param. parameters

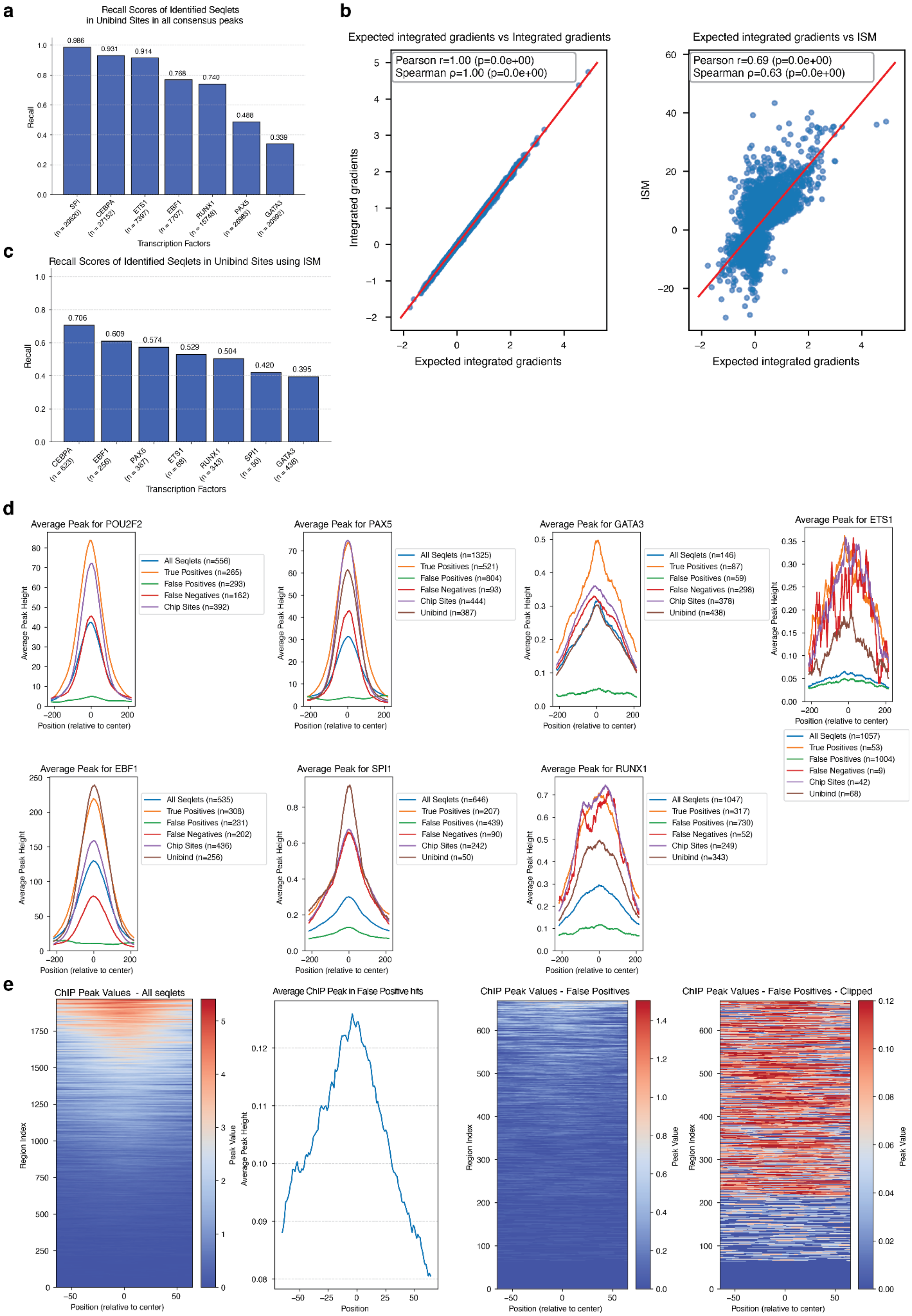
a
Recall Scores of Identified Seqlets
in Unibind Sites in all consensus peaks
Recall
0.986
0.931
0.914
0.768
0.740
0.488
0.339
SPI (n = 29620)
CEBPA (n = 27152)
ETS1 (n = 7397)
EBF1 (n = 7707)
RUNX1 (n = 15748)
PAX5 (n = 28983)
GATA3 (n = 20992)
Transcription Factors
b
Expected integrated gradients vs Integrated gradients
Pearson r=1.00 (p=0.0e+00)
Spearman ρ=1.00 (p=0.0e+00)
Integrated gradients
Expected integrated gradients
Expected integrated gradients vs ISM
Pearson r=0.69 (p=0.0e+00)
Spearman ρ=0.63 (p=0.0e+00)
ISM
Expected integrated gradients
c
Recall Scores of Identified Seqlets in Unibind Sites using ISM
Recall
0.706
0.609
0.574
0.529
0.504
0.420
0.395
CEBPA (n = 623)
EBF1 (n = 256)
PAX5 (n = 387)
ETS1 (n = 68)
RUNX1 (n = 343)
SPI1 (n = 50)
GATA3 (n = 438)
Transcription Factors
d
Average Peak for POU2F2
All Seqlets (n=556)
True Positives (n=265)
False Positives (n=293)
False Negatives (n=162)
Chip Sites (n=392)
Average Peak for PAX5
All Seqlets (n=1325)
True Positives (n=521)
False Positives (n=804)
False Negatives (n=93)
Chip Sites (n=444)
Unibind (n=387)
Average Peak for GATA3
All Seqlets (n=146)
True Positives (n=87)
False Positives (n=59)
False Negatives (n=298)
Chip Sites (n=378)
Unibind (n=438)
Average Peak for ETS1
All Seqlets (n=1057)
True Positives (n=53)
False Positives (n=1004)
False Negatives (n=9)
Chip Sites (n=42)
Unibind (n=68)
Average Peak for EBF1
All Seqlets (n=535)
True Positives (n=308)
False Positives (n=231)
False Negatives (n=202)
Chip Sites (n=436)
Unibind (n=256)
Average Peak for SPI1
All Seqlets (n=646)
True Positives (n=207)
False Positives (n=439)
False Negatives (n=90)
Chip Sites (n=242)
Unibind (n=50)
Average Peak for RUNX1
All Seqlets (n=1047)
True Positives (n=317)
False Positives (n=730)
False Negatives (n=52)
Chip Sites (n=249)
Unibind (n=343)
Average Peak Height
Position (relative to center)
e
ChIP Peak Values - All seqlets
Average ChIP Peak in False Positive hits
ChIP Peak Values - False Positives
ChIP Peak Values - False Positives - Clipped
Region Index
Peak Value
Average Peak Height
Position (relative to center)
Position

Figure S5. Additional UniBind and ChIP-seq comparisons.

(**a**) Recall of UniBind sites identified by DeepPBMC in all consensus peaks. (**b**) Comparison of per-nucleotide contribution scores for different explanation methods of 171 in vivo validated mouse cortical enhancers[17] using the DeepBICCN2 model. (**c**) Recall of found Unibind hits for a set of different TFs in the top 1000 most specific regions per PBMC cell type using ISM (bottom). (**d**) The aggregated peak heights of all identified seqlets, true positive seqlets, false positive seqlets, false negative ChIP peaks, and all ChIP-seq and UniBind hits in the top 1,000 regions per cell type are shown per TF. (**e**) Carrot plot of ChIP-seq peak heights of all identified CEBPA seqlets (n=1,968 seqlets) (left), the aggregated ChIP-seq peak for all false positive CEBPA seqlets (n=669 seqlets) (second to left), carrot plot of ChIP-seq peak heights of false positive CEBPA seqlets (n=669 seqlets) (second to right) and a clipped carrot plot of ChIP-seq peak heights of false positive CEBPA seqlets.

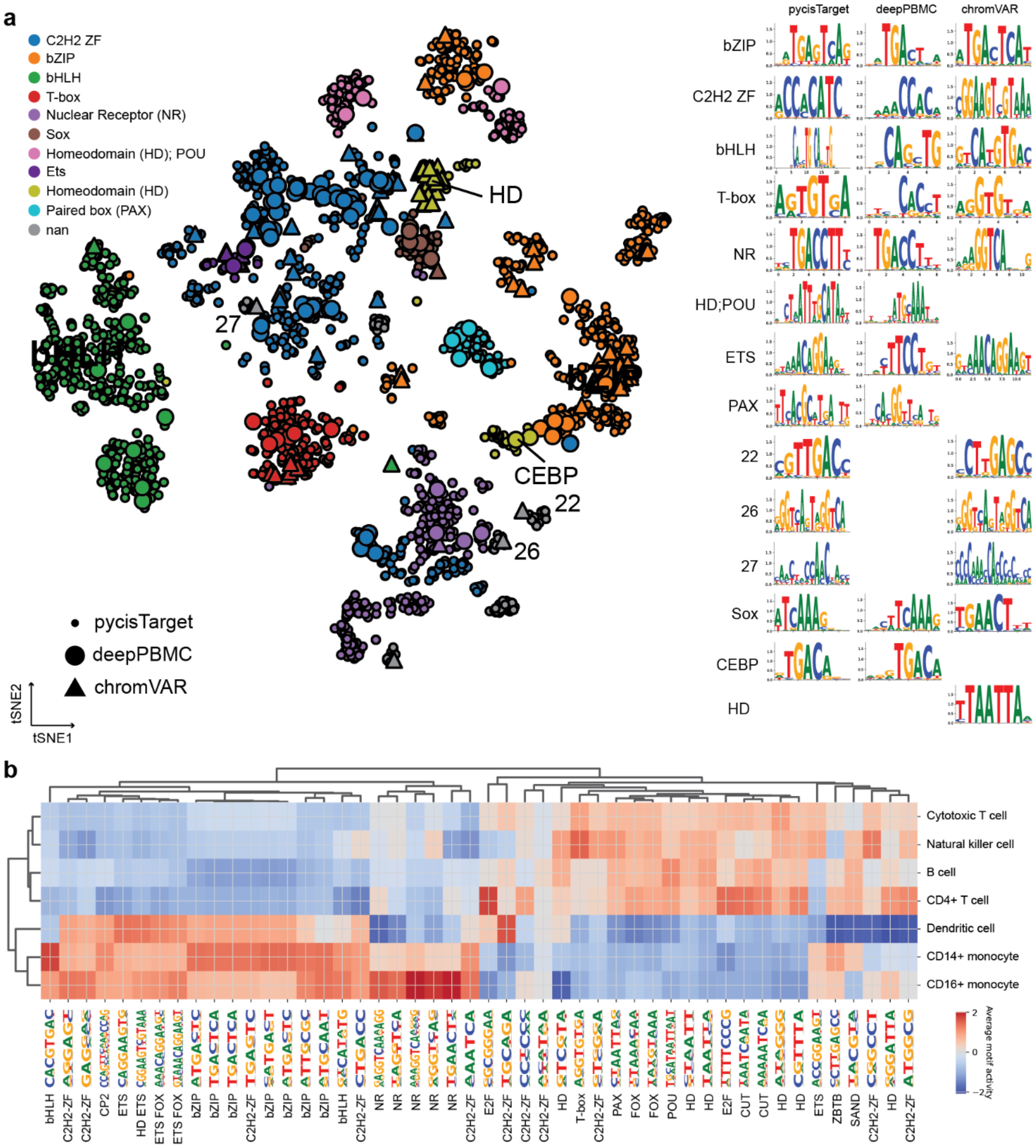

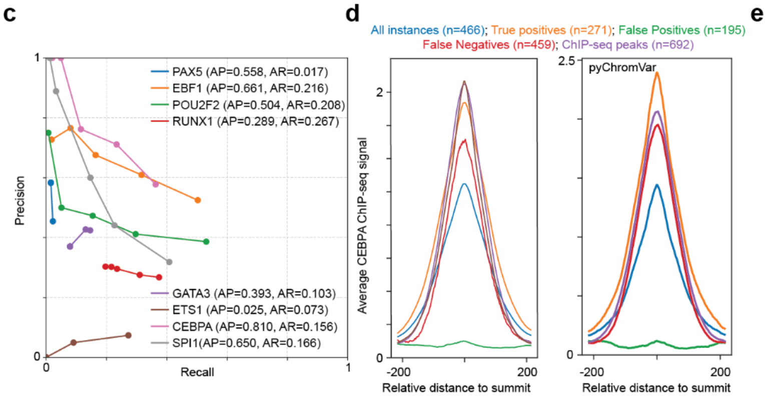

e

	CREsted		pyChromVar	
TF	Precision	Recall	Precision	Recall
PAX5	0.393	**0.849**	**0.441**	0.034
EBF1	**0.568**	**0.601**	0.525	0.504
POU2F2	**0.473**	**0.619**	0.388	0.532
RUNX1	**0.303**	**0.859**	0.260	0.377
GATA3	**0.596**	**0.226**	0.404	0.149
ETS1	0.050	**0.855**	**0.073**	0.273
CEBPA	**0.660**	**0.946**	0.582	0.371
SPI1	**0.320**	**0.697**	**0.320**	0.412
Average	**0.420**	**0.707**	0.374	0.332

Figure S6. Motif identification benchmark comparing CREsted and motif enrichment analysis tools on PBMC data.

(**a**) Left: tSNE of motifs found enriched using pycisTarget (n=2,100 motifs) and chromVAR (n=125 motifs) and patterns found using TF-MoDISco based on contribution scores from deepPBMC (n=139 patterns). Motifs and patterns are clustered based on their motif similarity calculated using TomTom. And annotated at cluster level based on the SCENIC+ motif-to-TF annotation database. Right: Representative motif for each TF family/cluster. (**b**) Heatmap of average motif activity per cell type calculated using chromVAR. (**c**) Precision-recall curve on comparing pyChromVAR identified seqlets and ChIP-seq peaks in the top 1,000 peaks for the corresponding cell type. Thresholding is done on the 'motif match' score. Average precision (AP) and recall (AR) over the thresholds are indicated in the legend. (**d**) Average ChIP peak height of different sets of proposed TFBS for CEBPA in the top 1,000 most-specific CD14+ monocytes identified through CREsted (left) and pyChromVar (right). (**e**) Precision-recall table for a set of TFs comparing CREsted and pyChromVar motif identification overlapping with ChIP-seq peaks at the lowest threshold in both settings.

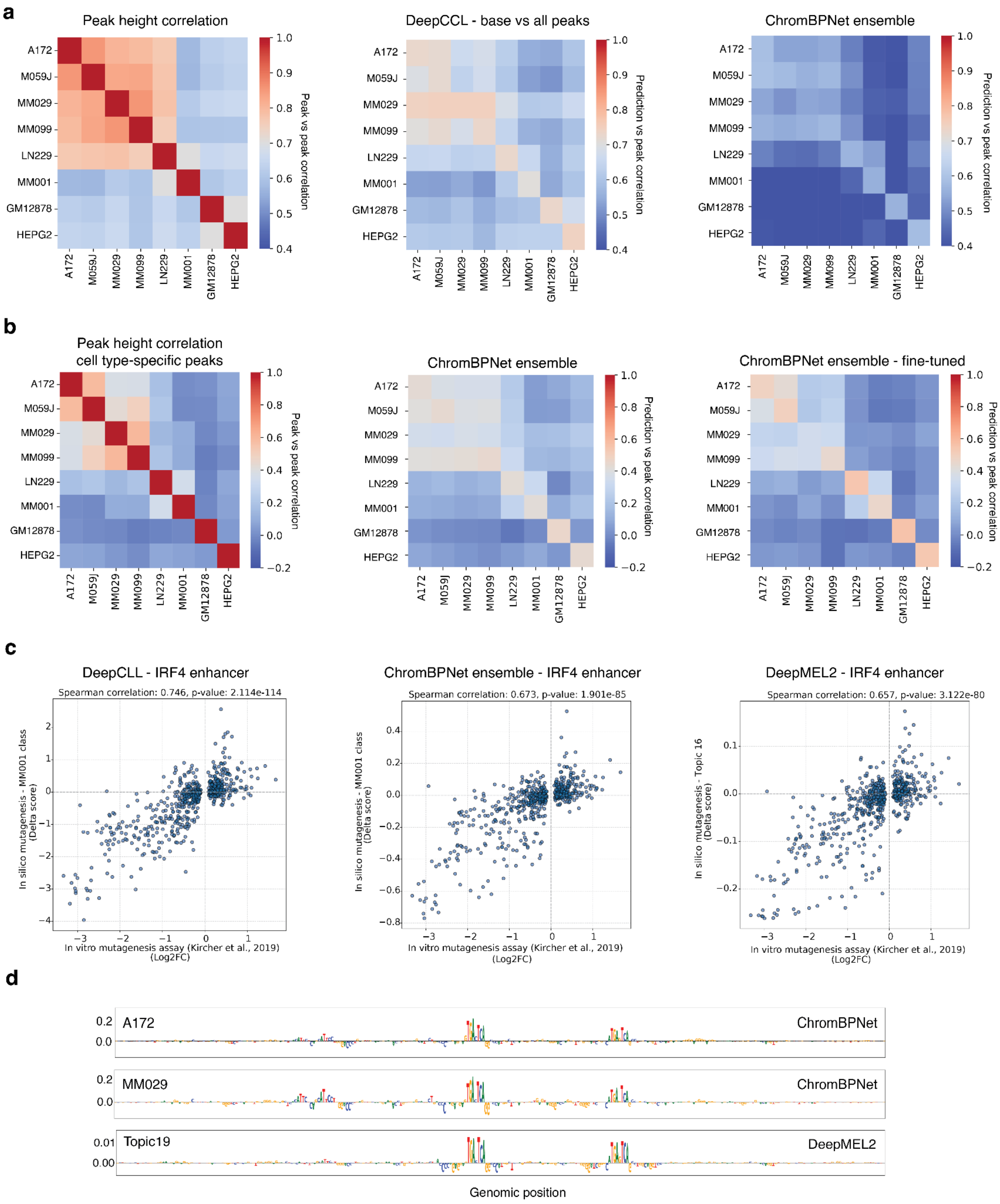
a
Peak height correlation
Peak vs peak correlation
DeepCCL - base vs all peaks
Prediction vs peak correlation
ChromBPNet ensemble
Prediction vs peak correlation
A172
M059J
MM029
MM099
LN229
MM001
GM12878
HEPG2
b
Peak height correlation
cell type-specific peaks
Peak vs peak correlation
ChromBPNet ensemble
Prediction vs peak correlation
ChromBPNet ensemble - fine-tuned
Prediction vs peak correlation
c
DeepCLL - IRF4 enhancer
Spearman correlation: 0.746, p-value: 2.114e-114
In silico mutagenesis - MM001 class
(Delta score)
In vitro mutagenesis assay (Kircher et al., 2019)
(Log2FC)
ChromBPNet ensemble - IRF4 enhancer
Spearman correlation: 0.673, p-value: 1.901e-85
In silico mutagenesis - MM001 class
(Delta score)
In vitro mutagenesis assay (Kircher et al., 2019)
(Log2FC)
DeepMEL2 - IRF4 enhancer
Spearman correlation: 0.657, p-value: 3.122e-80
In silico mutagenesis - Topic 16
(Delta score)
In vitro mutagenesis assay (Kircher et al., 2019)
(Log2FC)
d
A172
ChromBPNet
MM029
ChromBPNet
Topic19
DeepMEL2
Genomic position

Figure S7. DeepCCL, ChromBPNet and DeepMEL2 performance comparison.

(**a**) For the set of all test set regions (n=48,268 regions), the log-transformed peak height Pearson correlation across cell types (left), the Pearson correlation between the log-transformed DeepCLL base model predictions and peak values (middle) and the Pearson correlation between the combined ChromBPNet model predictions and peak values (right). (**b**) For the set of cell type-specific test set regions (n=27,020 regions), the log-transformed peak height Pearson correlation across cell types (left), the Pearson correlation between the log-transformed combined ChromBPNet model predictions and peak values (middle) and the Pearson correlation between the combined ChromBPNet model, fine-tuned on specific regions, predictions and peak values (right). (**c**) Scatter plot comparison between ISM and in vitro mutagenesis values[102] for the IRF4 enhancer (n=451 nucleotides) in MEL-classes (MM001 in DeepCCL and ChromBPNet, and Topic 19 in DeepMEL2). (**d**) Contribution scores from ChromBPNet and DeepMEL2 for the identified intronic *AXL* region (hg38 chr19:41,222,657-41,224,771, zoomed to center 300 bp).

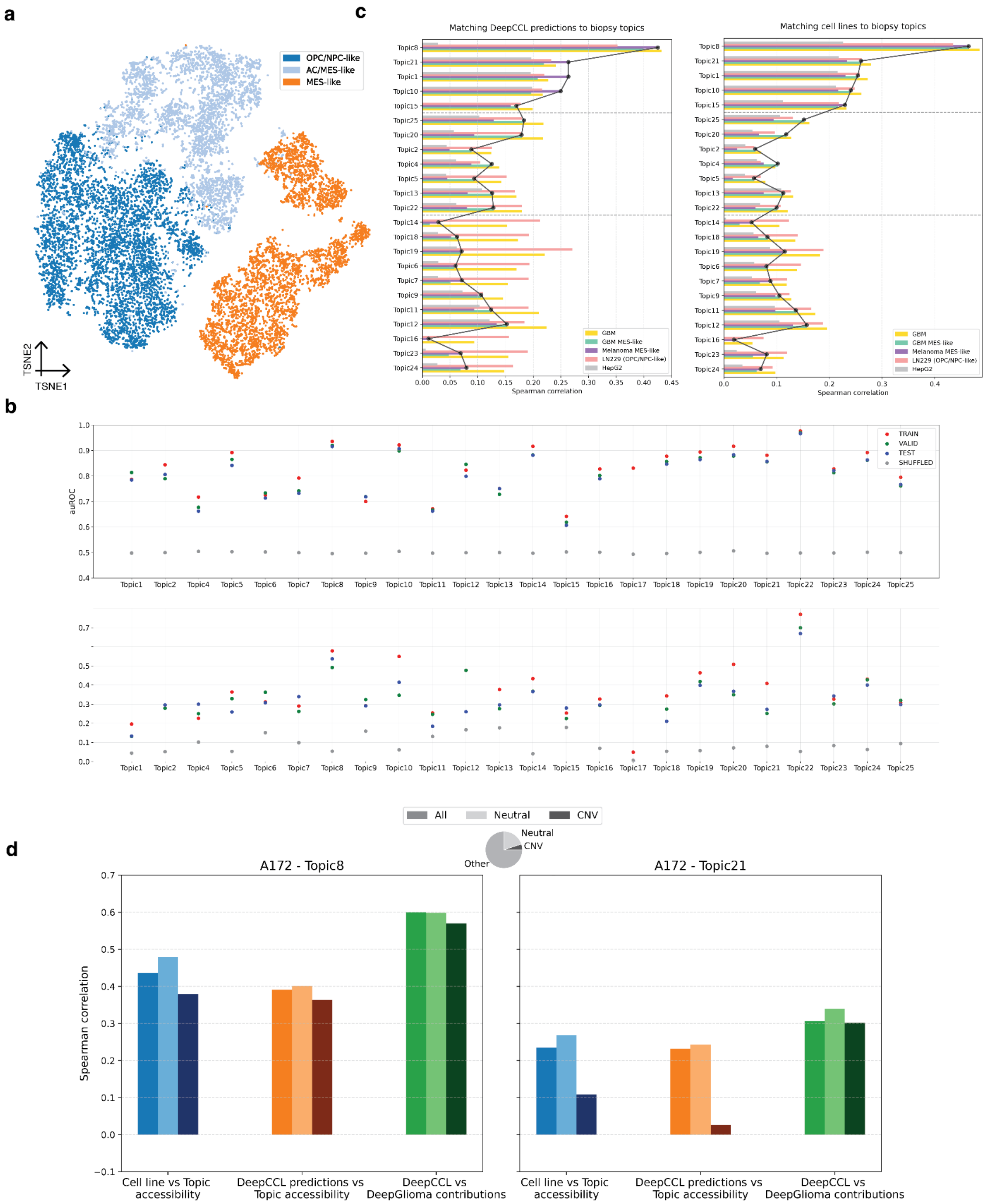

Figure S8. Comparison between DeepCCL cell lines and DeepGlioma topics.

(**a**) tSNE dimensionality reduction of 14,275 glioma cells manually annotated clusters for the Wang *et al*. glioma biopsy data. (**b**) Area under receiver operating characteristic curve (auROC) (top) and area under PR-curve (bottom) per topic for the different splits for the DeepGlioma model. (**c**) Spearman correlation between DeepCCL predictions / cell line accessibility and pseudobulked Topic bigwig files obtained from the pycisTopic analysis. GBM: average prediction/accessibility of

A172, M059J, and LN229. GBM MES-like: average prediction/accessibility of A172 and M059J. Melanoma MES-like: average prediction/accessibility of MM029 and MM099. Line plot illustrates the max MES-like correlation across the GBM MES-like and Melanoma MES-like bars. (**d**) Spearman correlations are shown for three comparisons across all regions (n=10,000/1000/1000 regions for accessibility, Topic 8 contributions, and Topic 21 contributions comparisons, respectively; light colors), neutral regions (n=1925/181/215 regions for accessibility, Topic 8 contributions, and Topic 21 contributions comparisons, respectively; medium colors), and CNV regions (n=441/42/106 regions for accessibility, Topic 8 contributions, and Topic 21 contributions comparisons, respectively; dark colors). The three comparisons are: (1) A172 cell coverage accessibility vs. Topic accessibility, (2) DeepCCL A172 prediction scores vs. Topic accessibility, and (3) DeepCCL A172 contribution scores vs. DeepGlioma Topic contribution scores. The pie chart shows the relative proportion of All, Neutral, and CNV windows used in the analysis.

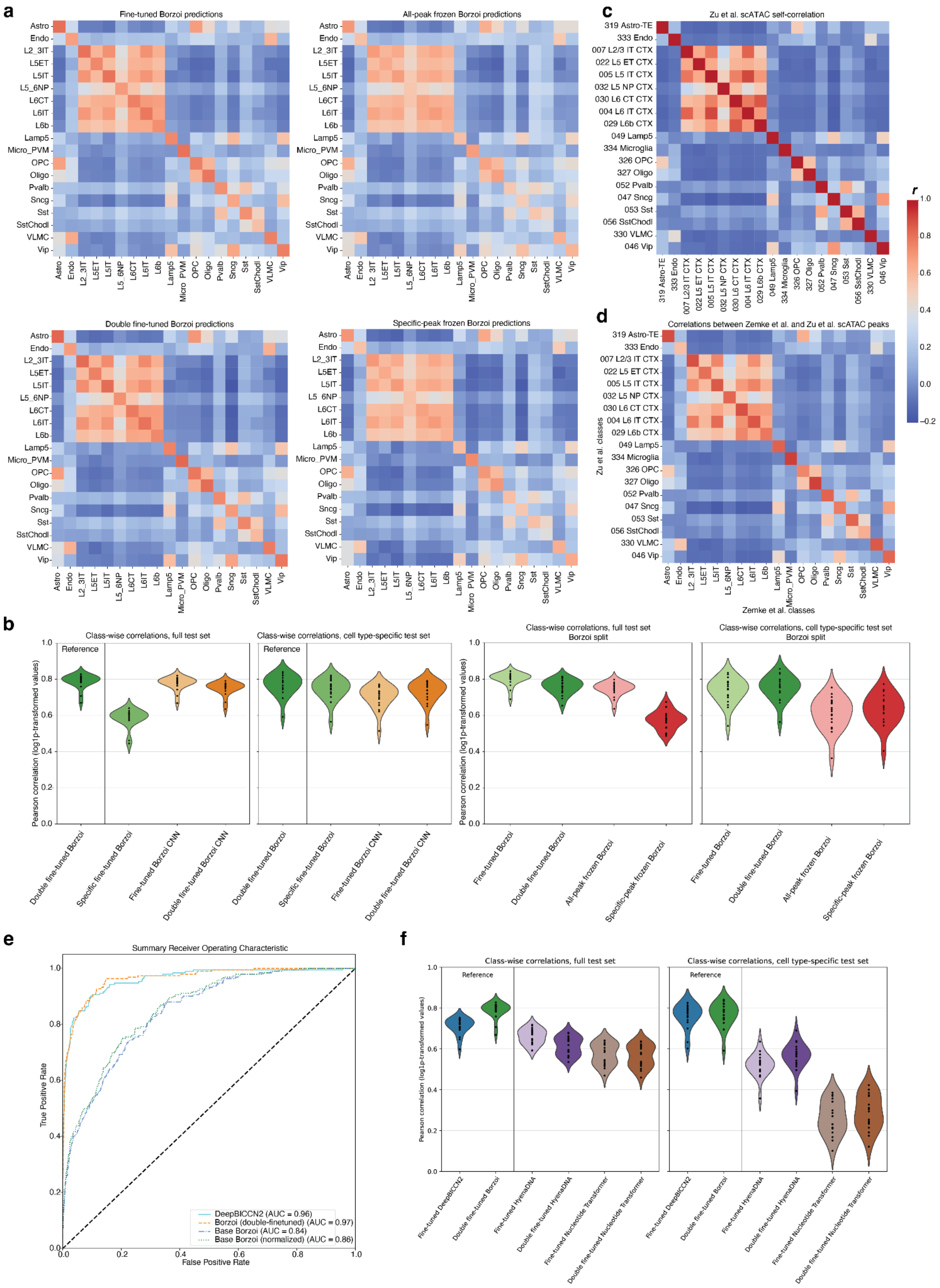
a
Fine-tuned Borzoi predictions
All-peak frozen Borzoi predictions
Double fine-tuned Borzoi predictions
Specific-peak frozen Borzoi predictions
Astro
Endo
L2_3IT
L5ET
L5IT
L5_6NP
L6CT
L6IT
L6b
Lamp5
Micro_PVM
OPC
Oligo
Pvalb
Sncg
Sst
SstChodl
VLMC
Vip
c
Zu et al. scATAC self-correlation
319 Astro-TE
333 Endo
007 L2/3 IT CTX
022 L5 ET CTX
005 L5 IT CTX
032 L5 NP CTX
030 L6 CT CTX
004 L6 IT CTX
029 L6b CTX
049 Lamp5
334 Microglia
326 OPC
327 Oligo
052 Pvalb
047 Sncg
053 Sst
056 SstChodl
330 VLMC
046 Vip
r
1.0
0.8
0.6
0.4
0.2
0.0
−0.2
d
Correlations between Zemke et al. and Zu et al. scATAC peaks
Zu et al. classes
Zemke et al. classes
b
Class-wise correlations, full test set
Class-wise correlations, cell type-specific test set
Class-wise correlations, full test set Borzoi split
Class-wise correlations, cell type-specific test set Borzoi split
Reference
Pearson correlation (log1p-transformed values)
Double fine-tuned Borzoi
Specific fine-tuned Borzoi
Fine-tuned Borzoi CNN
Double fine-tuned Borzoi CNN
Fine-tuned Borzoi
All-peak frozen Borzoi
Specific-peak frozen Borzoi
e
Summary Receiver Operating Characteristic
True Positive Rate
False Positive Rate
DeepBICCN2 (AUC = 0.96)
Borzoi (double-finetuned) (AUC = 0.97)
Base Borzoi (AUC = 0.84)
Base Borzoi (normalized) (AUC = 0.86)
f
Class-wise correlations, full test set
Class-wise correlations, cell type-specific test set
Reference
Pearson correlation (log1p-transformed values)
Fine-tuned DeepBICCN2
Double fine-tuned Borzoi
Fine-tuned HyenaDNA
Double fine-tuned HyenaDNA
Fine-tuned Nucleotide Transformer
Double fine-tuned Nucleotide Transformer

Figure S9. Performance of large transfer-learned models

(**a**) Heatmaps of Pearson correlations for log1p-transformed cell type-specific test set peaks (n=8,198 regions), analogous to Fig. 2c, for the predictions from the transfer learned models from Fig. 5b, comparing each cell type's peaks against each cell type's predictions. (**b**) Violin plots showing Pearson correlation values between the log1p-transformed test sets (all consensus peaks n=49,936 regions and cell type-specific peaks respectively n=8,198 regions) and corresponding predictions, calculated across the peaks for each cell type. (left) Comparison of the best-performing transfer learned model (Double fine-tuned Borzoi) with other transfer learning approaches (Specific fine-tuned: fine-tuning once, on specific peaks only; Borzoi CNN: identical fine-tuning steps as Fig. 5 approach, but using only the convolutional tower from the pre-trained Borzoi model) on the *r* per cell type (n=19 classes). (right) Correlation values per cell type (n=19 classes) for models trained and evaluated analogously to fig. 5b, but using a train/validation/test split based on the Borzoi folds instead (same model terminology from Fig. 5b). **(c)** Heatmap showing the self-correlation (Pearson correlations of log1p-transformed data) between selected Zu et al. pseudobulk scATAC classes, based on the cell type-specific set (n=8,198 regions). **(d)** Heatmap showing the correlations between Zu et al. and Zemke et al. scATAC pseudobulk classes, based on the cell-type specific set (n=8,198 regions). (**e**) Comparison of DeepBICCN2, base Borzoi (raw and normalized), and the double fine-tuned Borzoi model on the Zemke *et al.* mouse motor cortex data. ROC curve was made on the true and false positive rate of the specificity of the predictions on a set of 171 *in vivo* validated enhancers. (**f**) Pearson correlation metric for predicting cell type-specific chromatin accessibility profiles (n=8,198 regions) for fine-tuned CREsted model, fine-tuned Borzoi model and fine-tuned genomic language models.

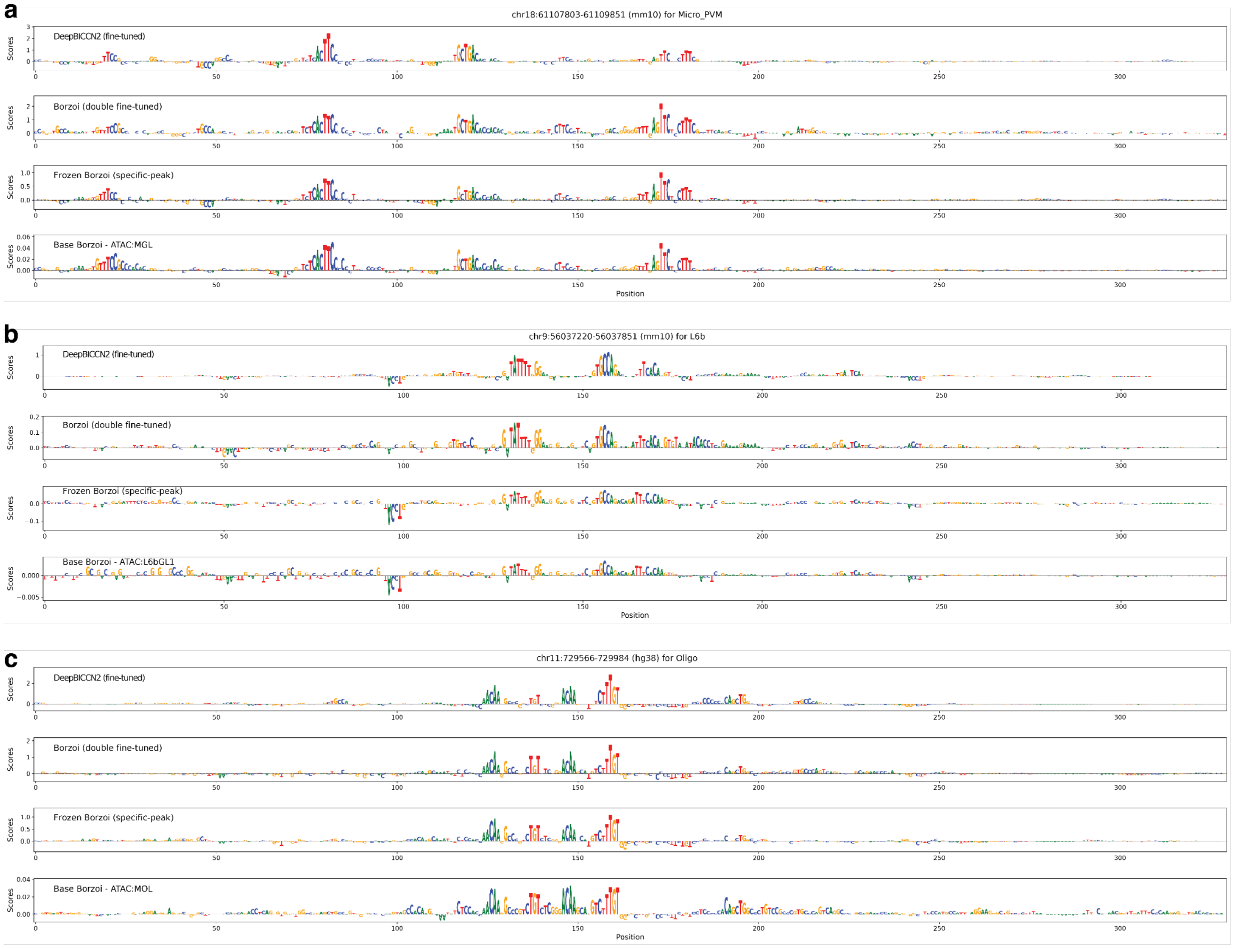

Figure S10. Transfer learning model contribution scores for additional validated enhancers.

DeepBICCN2, transfer learned Borzoi, and base Borzoi contribution scores for validated enhancers, calculated using expected integrated gradients. **(a)** Validated microglia enhancer[26] **(b-c)** Validated enhancers[17] from Fig. 2f not already shown in Fig. 5e.

Supplementary Notes

Note S1. Explanation and validation of peak-scaling in CREsted.

We rely on comparing peak heights across cell types, thus we need to ensure those values are scaled appropriately. CPM-normalized tracks are only comparable if two tracks have a similar number of peaks, but when one of them has more peaks than the other, values will be lower overall because of the larger total amount of fragments used in to normalize the track (fig. S1A). We therefore apply a min-max normalization, by looking at the top peaks per cell type (configurable by user, default top 1%), filtering out peaks that are non-specific (Gini index less than the mean of Gini indices of all peaks minus one standard deviation) with the aim of only retaining strong, housekeeping constitutive peaks. We then calculate the average peak height of the strongest peaks per cell type, and obtain scalars per cell type which result in having the same average value per cell type. Finally, all peak values per cell type are updated by these cell type-specific peak scalars. This approach was first described in Johansen & Kempynck *et al*. 2025[16].

As an example, we show the peak height distributions of the promoters of *Canx* and *Zfp106*, two examples that are among the set of the 22 most stable promoters (fig. S1b). This highlights that there is a substantial amount of variability in peak heights across cell types with a coefficient of variation (CV) of 0.45 and 0.40 respectively in CPM normalized peaks. Particularly, peaks from excitatory neurons are lower than those from non-neuronal cell types, with up to a threefold difference in Layer (L) 5 extra-telencephalic (ET) and microglia (Micro-PVM) peak heights. To further generalize these findings, we calculated the average accessibility over all types for all housekeeping gene promoters. While the average expression of all of these genes across cell types is indeed consistent (CV = 0.08) (fig. S1c), their average accessibility is highly variable (CV = 0.36). The variability in peak heights of these housekeeping promoters is indeed consistent with the peak-scaling scalars we identified through CREsted (fig. S1d). In other words, they follow the expected values needed for scaling the housekeeping promoters to the same value.

Note S2. Robustness analysis of CREsted models.

To further scrutinize the CREsted model training pipeline, we assessed the performance of CREsted models directly trained on cell type-specific peaks without pretraining on all consensus peaks. Such models have worse performance metrics than both fine-tuned and base models and have worse gene locus predictions (fig. S2a and c). We additionally investigated the effect of chromosomal train, validation and test split on model performance, and found consistent robustness over different splits (fig. S2a and b). Lastly, we doubled our peak set with a set of non-peak regions to assess whether this would increase general and non-peak predictive performance, but report no improvements (fig. S1g and h). Even within the set of consensus peaks, a large fraction of target values are near zero because most peaks are open only in a subset of all types, this seems to suffice and not necessitate the need of adding additional non-peak regions.

Note S3. Borzoi fine-tuning strategies

Shrinking the Borzoi model

Borzoi predicts chromatin accessibility, DNA binding, and gene expression for many biological samples across a large genomic window, taking 512k bp input sequences, and predicting binned signals for the core 192k bp. To adjust this track coverage-predicting model for use with transfer learning on individual peak regions, we change it in two ways: shrinking the model's input size to the size of a single region, and replacing the final layer to predict a vector of peak heights per region.

As the model bins to 128 bp internally, its new input size must be a multiple of 128 bp. We shrink these large models' input size to 2,048 bp to approximate the *DilatedCNN* architecture input size. To change the model output from multiple-bin predictions to predicting a single vector of peak heights, we first disable the cropping layer and remove the final class-specific head layer. To replace the final layer, we instead flatten the model's output embeddings and feed them to a single Dense layer with a Softplus activation, predicting the number of cell types desired. This results in a peak regression model benefitting from the weights of the original large pre-trained model.

Main models' performance on consensus and specific regions

For many models, a trade-off exists between performance on the full set of consensus peaks and on cell type-specific peaks (Figs. 2d, 4c, 5b). When evaluating the models trained on brain cortex data (Figs. 5b and S9a), all models trained on consensus peaks performed better on this full set of peaks than the cell type-specific finetuned models: out of the models further fine-tuned on cell type-specific peaks, however, fine-tuned Borzoi's performance on the full test set only dropped very slightly after double fine-tuning (0.80 to 0.78 mean r), while DeepBICCN2's performance drop-off is more substantial (0.79 to 0.71 mean r), potentially indicating a better capability of large pre-trained models like Borzoi to retain predictive capabilities for non cell type-specific peaks.

On the cell type-specific test set, the DeepBICCN2 and fine-tuned Borzoi models each realize a slight performance boost after the second round of fine-tuning (0.74 to 0.76 mean r and 0.75 to 0.78 mean r respectively; Fig. 5b). The frozen Borzoi models clearly perform worse than either fully trained model type (mean r of 0.61 and 0.63 respectively for the all peak and cell type-specific peak models). We additionally observed that fine-tuning solely on the cell type-specific peaks showed worse performance compared to the double fine-tuning approach (Fig. S9b).

We conclude that fine-tuning on Borzoi models requires full retraining, rather than training a single fully connected layer on the Borzoi embeddings; and that simple CREsted architectures with only 6M parameters and trained on 19 chromatin accessibility tracks match the performance of large models like Borzoi with 170M parameters, trained on 6230 regulatory and 3989 gene expression tracks.

Pre-training data leakage

Since the transfer learning was done with the train, validation, and test splits as the DeepBICCN2 model, it was not aligned with the original data split used to train Borzoi, which could result in artificial inflation of model performance metrics through data leakage.
To evaluate this, we re-distributed the regions into train, validation and test splits according to the Borzoi replicate's splits (using fold 4 as validation and fold 3 as test set, and the other folds as train set). Transfer learning on the regions with this distribution resulted in near-equivalent performance (Fig. S9b).

Alternative pre-trained architectures
Since the primary goal of the transformer layers in the Borzoi architecture is to integrate long-range interactions and the base CREsted architectures are solely CNN-based, we investigated whether fine-tuning a model solely based on Borzoi's CNN tower would reach similar performance. However, the fine-tuned Borzoi CNN-only models slightly underperformed the full-Borzoi models (fig. S9b), indicating the transformer and upsampling layers could contain useful prior information for fine-tuning on scATAC peak heights.

Note S4. Advantages of deep learning based enhancer modeling over classical motif enrichment/discovery analysis.

From the early 2000's, motif discovery and motif enrichment analysis have been used to model *cis*-regulatory elements[57,103–120] and current motif databases contain motifs for almost all transcription factors (TFs)[34]. Sequence-to-function deep learning models learn TF binding motifs *de novo* in order to make biologically relevant predictions. Indeed, all motifs learned by DeepPBMC match to known motifs obtained through motif enrichment analysis (Fig. S6a). The use of deep learning based enhancer modeling has, however, several advantages over classical motif enrichment analysis/discovery. These advantages include:

1. **Finding motif instances**. The discovery of enriched motifs in sets of co-accessible regions can also be performed with classical motif discovery methods. However, after a motif is found significantly enriched, the identification of the true positive instances of this motif, within a critical subset (or leading edge) of the input set, has always been a difficult challenge[77]. Sequence-to-function models, through seqlet calling, provide a solution to this problem. In addition, they provide motif instances before motif discovery, meaning that TF binding site (TFBS) predictions can be obtained even if not enough occurrences are found that would lead to a significant enrichment over the input set. For example, it has been shown that sequence-to-function models can be used to correctly interpret the effect of mutations to functioning enhancers. The mutations that affect enhancer activity significantly correspond to transcription factor binding sites and these are accurately (with high precision and recall) found by sequence-to-function models[10,13,25,121].
2. **Finding cell type-specific motifs *de novo***. Even though our current transcription factor motif databases are close to complete, for organisms that are well studied. For other, more rarely studied organisms that are evolutionarily distant from human, fly or mouse, the de novo discovery aspect might be more beneficial. Sequence-to-function models have been

used across many studies[9,13,24–26,122–126] for *de novo* motif discovery, especially making use of downstream TF-MoDISco analysis[39].

3. **Predicting the effect of non-coding variation.** Although non-coding variants can also be interpreted using position weight matrices, their predictive power is limited[127]. Sequence-to-function models might be more powerful in this regard[8,10,29].
4. **Transcription factor binding sites are seen in the context of other binding sites**. Enhancers consist of specific combinations of transcription factor binding sites. Because sequence-to-function models process full enhancer sequences at once, these contextual relationships can be learnt by the model. For example, Kim *et al.* Assessed the effect of heterotypic pairs of transcription factor binding sites and show that most pairs, according to the model, have a multiplicative effect[128]; Taskiran *et al.* showed that the introduction of ZEB2 binding sites in functional enhancers using a CNN brings down the in vitro activity. Adding Mamo repressor sites to Kenyon Cell enhancers abolishes enhancer activity in vivo[41]; and McAnany *et al*. developed a new tool that allows for the inference of the contribution of each nucleotide to the chromatin accessibility of each nucleotide of a chromatin accessibility peak showing that multiple TF binding sites can affect the chromatin accessibility of a single nucleotide[129].
5. **Learning syntactic rules**. Syntactic rules like distance dependent relationships between individual transcription factor binding sites, binding affinity and copy number of individual binding sites might be important for cell type-specific enhancer activity (although these rules are properly not strict (i.e., soft syntax[2])). Sequence-to-function deep learning models have the potential to learn such rules. For example, Avsec *et al*. identified distance dependent cooperativity for TF binding of Oct4, Sox2, Nanog and Klf4 in embryonic stem cells[9] and de Almeida *et al*. and Taskiran *et al.* identified distance dependent effects of TF pairs on enhancer activity[14,41].
6. **Scoring of unseen sequences/genomes**. As we showed in figure 2e, sequence-to-function models can be used to score the genome of other species and are able to accurately recover chromatin accessibility peaks. In the same line, Minnoye and Taskiran *et al*. identified melanoma enhancers across species using DeepMEL[13]; Zemke *et al.* used one species' genome as test set to evaluate the performance of models trained on other species[42]; and Hecker and Kempynck *et al*. identified conserved cell types across mammalian and bird brains[26]. Repeating this same analysis with position weight matrices alone would result in large excesses of false positive predictions[77].
7. **Design of cell type-specific enhancers**. Sequence-to-function deep learning models can be used effectively as biological oracles in order to design cell type-specific enhancers[12,41,130–133], as we show for example in figure 6. Without the use of deep learning models this endeavor has proven unfruitful[134].

Note S5. Discussion on zebrafish enhancer design.

We designed enhancers for somatic muscle, cardiac muscle, and endothelial cells with predicted accessibility within the range of genomic enhancers. These single cell type synthetic enhancers had a high success rate with all somatic and cardiac muscle enhancers, and one out of three

endothelial enhancers being specifically active in the target cell type. This is especially remarkable given the high similarity of the enhancer codes of cardiac and somatic muscle cells (Fig. 6o). Previous work by Gosai *et al.*[131] also validated synthetically designed sequences in zebrafish, targeting neurons and the liver. DeepZebrafish adds scale and resolution to these experiments, as we present the possibility of designing enhancers, targeting single and dual cell types, at cell type-specific resolution for the entire organism during development.

As a follow-up of our earlier work in the Drosophila brain[41], we designed dual cell type enhancers (somatic and cardiac muscle), with overall more mosaic reporter expression. While designing these dual cell type enhancers, we designed multiple different sets that were suboptimal for one of the two cell types with the intent of modulating the level of GFP expression. We did observe an effect of the predicted level of expression, in the sense that enhancers that were predicted to have a higher level in somatic muscle cells compared to cardiac muscle cells, indeed labeled more somatic muscle cells compared to enhancers that were predicted to have a higher level of expression in cardiac muscle compared to somatic muscle cells. However, enhancers that were predicted to have equal levels of expression in both cell types labeled fewer somatic muscle cells in fewer embryos, compared to the enhancers that were designed to be stronger in somatic muscle cells (vis-à-vis cardiac muscle cells) even though the predicted level of somatic muscle cell chromatin accessibility of both enhancer sets is equal. This was an unexpected finding that warrants further investigation, as precise control over cargo expression levels in enhancer reporter assays holds significant relevance for disease-related gene therapy applications[135]. Of note, full quantification of expression levels may require the use of stable transgenics. In contrast, in our experiments, the observed reporter expression results from both the transient plasmid and the integrated enhancer.

Supplemental References

102. Kircher, M. *et al.* Saturation mutagenesis of twenty disease-associated regulatory elements at single base-pair resolution. *Nat. Commun.* **10**, 3583 (2019).

103. Van Helden, J., Andr□, B. & Collado-Vides, J. A web site for the computational analysis of yeast regulatory sequences. *Yeast* **16**, 177–187 (2000).

104. Frith, M. C., Hansen, U. & Weng, Z. Detection of *cis* -element clusters in higher eukaryotic DNA. *Bioinformatics* **17**, 878–889 (2001).

105. Frith, M. C. Statistical significance of clusters of motifs represented by position specific scoring matrices in nucleotide sequences. *Nucleic Acids Res.* **30**, 3214–3224 (2002).

106. Frith, M. C. Cluster-Buster: finding dense clusters of motifs in DNA sequences. *Nucleic Acids Res.* **31**, 3666–3668 (2003).

107. Aerts, S. Toucan: deciphering the cis-regulatory logic of coregulated genes. *Nucleic Acids Res.* **31**, 1753–1764 (2003).

108. Coessens, B. INCLUSive: a web portal and service registry for microarray and regulatory sequence analysis. *Nucleic Acids Res.* **31**, 3468–3470 (2003).

109. Aerts, S., Van Loo, P., Moreau, Y. & De Moor, B. A genetic algorithm for the detection of new *cis* -regulatory modules in sets of coregulated genes. *Bioinformatics* **20**, 1974–1976 (2004).

110. Van Helden, J. Metrics for comparing regulatory sequences on the basis of pattern counts. *Bioinformatics* **20**, 399–406 (2004).

111. Aerts, S., Van Helden, J., Sand, O. & Hassan, B. A. Fine-Tuning Enhancer Models to Predict Transcriptional Targets across Multiple Genomes. *PLoS ONE* **2**, e1115 (2007).

112. Thomas-Chollier, M. *et al.* RSAT: regulatory sequence analysis tools. *Nucleic*

Acids Res. **36**, W119–W127 (2008).

113. Grant, C. E., Bailey, T. L. & Noble, W. S. FIMO: scanning for occurrences of a given motif. *Bioinformatics* **27**, 1017–1018 (2011).

114. Bailey, T. L. *et al.* MEME SUITE: tools for motif discovery and searching. *Nucleic Acids Res.* **37**, W202–W208 (2009).

115. Herrmann, C., Van De Sande, B., Potier, D. & Aerts, S. i-cisTarget: an integrative genomics method for the prediction of regulatory features and cis-regulatory modules. *Nucleic Acids Res.* **40**, e114–e114 (2012).

116. Imrichová, H., Hulselmans, G., Kalender Atak, Z., Potier, D. & Aerts, S. i-cisTarget 2015 update: generalized cis-regulatory enrichment analysis in human, mouse and fly. *Nucleic Acids Res.* **43**, W57–W64 (2015).

117. Verfaillie, A., Imrichova, H., Janky, R. & Aerts, S. iRegulon and i-cisTarget: Reconstructing Regulatory Networks Using Motif and Track Enrichment. *Curr. Protoc. Bioinforma.* **52**, (2015).

118. Heinz, S. *et al.* Simple Combinations of Lineage-Determining Transcription Factors Prime cis-Regulatory Elements Required for Macrophage and B Cell Identities. *Mol. Cell* **38**, 576–589 (2010).

119. Van Heeringen, S. J. & Veenstra, G. J. C. GimmeMotifs: a *de novo* motif prediction pipeline for ChIP-sequencing experiments. *Bioinformatics* **27**, 270–271 (2011).

120. Bruse, N. & Heeringen, S. J. V. GimmeMotifs: an analysis framework for transcription factor motif analysis. Preprint at https://doi.org/10.1101/474403 (2018).

121. Kosicki, M. *et al.* In vivo mapping of mutagenesis sensitivity of human enhancers. *Nature* https://doi.org/10.1038/s41586-025-09182-w (2025) doi:10.1038/s41586-025-09182-w.

122. Sahu, B. *et al.* Sequence determinants of human gene regulatory elements. *Nat.*

Genet. **54**, 283–294 (2022).

123. Brennan, K. J. *et al.* Chromatin accessibility in the Drosophila embryo is determined by transcription factor pioneering and enhancer activation. *Dev. Cell* **58**, 1898-1916.e9 (2023).

124. Mannens, C. C. A. *et al.* Chromatin accessibility during human first-trimester neurodevelopment. *Nature* 1–8 (2024) doi:10.1038/s41586-024-07234-1.

125. Nair, S. *et al.* Transcription factor stoichiometry, motif affinity and syntax regulate single-cell chromatin dynamics during fibroblast reprogramming to pluripotency. Preprint at https://doi.org/10.1101/2023.10.04.560808 (2023).

126. Cochran, K. *et al.* Dissecting the cis-regulatory syntax of transcription initiation with deep learning. Preprint at https://doi.org/10.1101/2024.05.28.596138 (2024).

127. Yan, J. *et al.* Systematic analysis of binding of transcription factors to noncoding variants. *Nature* **591**, 147–151 (2021).

128. Kim, D. S. *et al.* The dynamic, combinatorial cis-regulatory lexicon of epidermal differentiation. *Nat. Genet.* **53**, 1564–1576 (2021).

129. McAnany, C. E. *et al.* PISA: a versatile interpretation tool for visualizing cis-regulatory rules in genomic data. Preprint at https://doi.org/10.1101/2025.04.07.647613 (2025).

130. de Almeida, B. P. *et al.* Targeted design of synthetic enhancers for selected tissues in the Drosophila embryo. *Nature* **626**, 207–211 (2024).

131. Gosai, S. J. *et al.* Machine-guided design of cell-type-targeting cis-regulatory elements. *Nature* **634**, 1211–1220 (2024).

132. Castillo-Hair, S. M. *et al.* Programming human cell type-specific gene expression via an atlas of AI-designed enhancers. Preprint at https://doi.org/10.1101/2025.09.30.679565 (2025).

133. Yin, C. *et al.* Iterative deep learning design of human enhancers exploits

condensed sequence grammar to achieve cell-type specificity. *Cell Syst.* **16**, 101302 (2025).

134. Vincent, B. J., Estrada, J. & DePace, A. H. The appeasement of Doug: a synthetic approach to enhancer biology. *Integr. Biol.* **8**, 475–484 (2016).

135. Matharu, N. & Ahituv, N. Modulating gene regulation to treat genetic disorders. *Nat. Rev. Drug Discov.* **19**, 757–775 (2020).
