# Supplementary material for: CREsted: modeling genomic and synthetic cell-type-specific enhancers across tissues and species
Source: Nat Methods. 2026 Apr 2;23(5):946–59. doi: 10.1038/s41592-026-03057-2 (PMC13167471; doi:10.1038/s41592-026-03057-2)
Supplement: Supplementary file 2 — Reporting Summary [file 41592_2026_3057_MOESM2_ESM.pdf]

nature portfolio

Corresponding author(s): Stein Aerts

Last updated by author(s): Nov 28, 2025

# Reporting Summary

Nature Portfolio wishes to improve the reproducibility of the work that we publish. This form provides structure for consistency and transparency in reporting. For further information on Nature Portfolio policies, see our Editorial Policies and the Editorial Policy Checklist.

## Statistics

For all statistical analyses, confirm that the following items are present in the figure legend, table legend, main text, or Methods section.

| n/a | Confirmed | |
|---|---|---|
| ☐ | ☒ | The exact sample size (*n*) for each experimental group/condition, given as a discrete number and unit of measurement |
| ☒ | ☐ | A statement on whether measurements were taken from distinct samples or whether the same sample was measured repeatedly |
| ☐ | ☒ | The statistical test(s) used AND whether they are one- or two-sided<br>*Only common tests should be described solely by name; describe more complex techniques in the Methods section.* |
| ☐ | ☒ | A description of all covariates tested |
| ☐ | ☒ | A description of any assumptions or corrections, such as tests of normality and adjustment for multiple comparisons |
| ☐ | ☒ | A full description of the statistical parameters including central tendency (e.g. means) or other basic estimates (e.g. regression coefficient) AND variation (e.g. standard deviation) or associated estimates of uncertainty (e.g. confidence intervals) |
| ☐ | ☒ | For null hypothesis testing, the test statistic (e.g. *F*, *t*, *r*) with confidence intervals, effect sizes, degrees of freedom and *P* value noted<br>*Give P values as exact values whenever suitable.* |
| ☒ | ☐ | For Bayesian analysis, information on the choice of priors and Markov chain Monte Carlo settings |
| ☒ | ☐ | For hierarchical and complex designs, identification of the appropriate level for tests and full reporting of outcomes |
| ☐ | ☒ | Estimates of effect sizes (e.g. Cohen's *d*, Pearson's *r*), indicating how they were calculated |

*Our web collection on statistics for biologists contains articles on many of the points above.*

## Software and code

Policy information about availability of computer code

| | |
|---|---|
| Data collection | *Provide a description of all commercial, open source and custom code used to collect the data in this study, specifying the version used OR state that no software was used.* |
| Data analysis | The CREsted package is available at https://github.com/aertslab/CREsted and https://crested.readthedocs.io and is stored at https://zenodo.org/records/15045960. All computational analyses for the main figures can be found in https://github.com/aertslab/CREsted-paper and are stored at https://zenodo.org/records/17791384. Following packages were used for data analyses, this information is also available in the key resource table accompanying this manuscript (https://zenodo.org/records/17791463). CREsted v. 1.4.0, pybigtools v. 0.2.0, ChromBPNet v.1.0, anndata v.0.11.3, Enformer, Borzoi, Keras v.3.0, TensorFlow v.2.19.0, PyTorch v.2.6.0, tfmodisco-lite v.2.2.1, tangermeme v.0.4.0, gReLU v. 1.0.3, SnapATAC2 v. 2.6.4, Harmony v. 0.0.10, statsmodels v0.14.4, Scipy v1.16, Python Programming Language v3, NIS-Elements, SCENIC+ v.1.0a, scatac_fragment_tools v.0.1.4, TF-MInDi v.1.0.0, pycisTarget v1.1, memesuite-lite v.0.2, scanpy v.1.11.4, pyChromVAR v.0.0.4, epiAneufinder v1.1.3, HyenaDNA, Nucleotide Transformer, Transformers v.4.54.1, PyTorch Lightning v.2.5.2, create_cisTarget_databases, WiggleTools v.1.2.11, MACS v2 and v3, pycisTopic v.2 and NGSCheckMate v.1.0.1. |

For manuscripts utilizing custom algorithms or software that are central to the research but not yet described in published literature, software must be made available to editors and reviewers. We strongly encourage code deposition in a community repository (e.g. GitHub). See the Nature Portfolio guidelines for submitting code & software for further information.

## Data

Policy information about availability of data

All manuscripts must include a data availability statement. This statement should provide the following information, where applicable:

- Accession codes, unique identifiers, or web links for publicly available datasets
- A description of any restrictions on data availability
- For clinical datasets or third party data, please ensure that the statement adheres to our policy

Analysis data required for reproducing the findings in this manuscript is available at https://resources.aertslab.org/CREsted/manuscript_data/. All CREsted models developed in this paper, and other legacy models, can be loaded through crested.get_model, or downloaded directly from https://resources.aertslab.org/CREsted/. All raw and processed sequencing data generated in this study have been deposited in NCBI's Gene Expression Omnibus and are accessible through GEO Series accession number GSE292617. This includes the OmniATAC-seq data of three human glioblastoma cell lines, namely A172, M059J, and LN229. Raw imaging data of the zebrafish enhancer reporter assays is available on EBI Biostudies using doi 10.6019/S-BIAD1962. The key resources table listing all resources needed to reproduce the results of this manuscript is available on Zenodo using the following doi: https://doi.org/10.5281/zenodo.17232414. Following publicly available datasets were used. Mouse cortex dataset (Zemke et al.) downloaded from GEO ( GSE229169); Human PBMC dataset (De Rop et al.) downloaded from GEO (GSE194028); Zebrafish developmental dataset (sun et al.) downloaded from GEO (GSE243256); Dataset of 100 enhancers tested in Zebrafish (Supplemental table of https://doi.org/10.1038/s41556-024-01449-0); Mouse brain pseudobulk BigWig (zu et al) downloaded from GEO (GSE246791); PAX5 ChIP-seq peaks downloaded from Encode (ENCFF827VVQ); PAX5 ChIP-seq B cells - BigWig track downloaded from Encode ( ENCFF914QGY); EBF1 ChIP-seq B cells - peaks downloaded from Encode (ENCFF895MHN); EBF1 ChIP-seq B cells - BigWig track downloaded form Encode (EBF1 ChIP-seq B cells - BigWig track); POU2F2 ChIP-seq B cells - peaks downloaded from Encode (ENCFF934JFA); POU2F2 ChIP-seq B cells - BigWig downloaded from Encode (ENCFF803HIP); GATA3 ChIP-seq CD4+ T cells - peaks & BigWig downloaded from chip-atlas (SRX4705120); RUNX1 ChIP-seq CD4+ T cells - peaks & BigWig downloaded from chip-atlas (SRX1492212); ETS1 ChIP-seq CD4+ T cells - peaks & BigWig downloaded from chip-atlas (SRX015825); CEBPA ChIP-seq CD14+ monocytes - peaks & BigWig downloaded from chip-atlas (SRX097095); SPI1 ChIP-seq CD14+ monocytes - peaks & BigWig downloaded from chip-atlas (SRX4001818); PAX5 ChIP-seq direct targets B cells (https://unibind.uio.no/factor/ENCSR000BHD.GM12878_female_B-cells_lymphoblastoid_cell_line.PAX5); EBF1 ChIP-seq direct targets B cells (https://unibind.uio.no/factor/ENCSR000DZQ.GM12878_female_B-cells_lymphoblastoid_cell_line.EBF1/); GATA3 ChIP-seq direct targets CD4+ T cells (https://unibind.uio.no/factor/GSE76181.Jurkat_T-cells.GATA3/); RUNX1 ChIP-seq direct targets CD4+ T cells (https://unibind.uio.no/factor/GSE76181.Jurkat_T-cells.RUNX1/); ETS1 ChIP-seq direct targets CD4+ T cells( https://unibind.uio.no/factor/EXP000299.Jurkat_E6_1_T-cells.ETS1/); CEBPA ChIP-seq direct targets CD14+ monocytes (https://unibind.uio.no/factor/EXP000946.U937_adult_acute_monocytic_leukemia.CEBPA/); SPI1 ChIP-seq direct targets CD14+ monocytes (https://unibind.uio.no/factor/EXP047756.MDMmonocyte_derived_macrophages.SPI1/); Human genome (hg38- https://hgdownload.cse.ucsc.edu/goldenPath/hg38/bigZips/); Mouse genome (mm10- https://hgdownload.cse.ucsc.edu/goldenPath/mm10/bigZips/)l; Zebrafish genome (danRer11 - https://hgdownload.cse.ucsc.edu/goldenPath/danRer11/bigZips/).

## Research involving human participants, their data, or biological material

Policy information about studies with human participants or human data. See also policy information about sex, gender (identity/presentation), and sexual orientation and race, ethnicity and racism.

| | |
|---|---|
| Reporting on sex and gender | NA |
| Reporting on race, ethnicity, or other socially relevant groupings | NA |
| Population characteristics | NA |
| Recruitment | NA |
| Ethics oversight | NA |

Note that full information on the approval of the study protocol must also be provided in the manuscript.

# Field-specific reporting

Please select the one below that is the best fit for your research. If you are not sure, read the appropriate sections before making your selection.

☒ Life sciences ☐ Behavioural & social sciences ☐ Ecological, evolutionary & environmental sciences

For a reference copy of the document with all sections, see nature.com/documents/nr-reporting-summary-flat.pdf

# Life sciences study design

All studies must disclose on these points even when the disclosure is negative.

| | |
|---|---|
| Sample size | No statistical method was used to predetermine sample size. Sample sizes were chosen based on the maximum amount of samples that were available for each analysis. For each analysis the sample size was sufficient to derive statistically meaningful results passing multiple testing procedures. |
| Data exclusions | No data was excluded in this study. |

| | |
|---|---|
| Replication | For the zebrafish enhancer reporter experiments multiple biological replicates were used. In general observed results could be replicated. We mentioned the number of replicates (and the number of positive samples) in the related figure. |
| Randomization | For the zebrafish enhancer reporter experiments the order in which enhancers were tested was randomized (i.e., enhancers designed for the same cell type were not all injected and imaged at the same experimental session). For the other analyses randomization was not relevant given that this is a study on a new machine learning method and no human judgment / subjective judgment was used to assess these models. Methods were compared using established and objective measurements. |
| Blinding | For the zebrafish enhancer reporter experiments enhancers were given a random name so that researchers were blind for the targetted cell type of each enhancer. For the other analyses performed in this study blinding was not relevant given that this is a study on a new machine learning method and no human judgment / subjective judgment was used to assess these models. Methods were compared using established and objective measurements. |

# Reporting for specific materials, systems and methods

We require information from authors about some types of materials, experimental systems and methods used in many studies. Here, indicate whether each material, system or method listed is relevant to your study. If you are not sure if a list item applies to your research, read the appropriate section before selecting a response.

## Materials & experimental systems

| n/a | Involved in the study | |
|---|---|---|
| ☒ | ☐ | Antibodies |
| ☐ | ☒ | Eukaryotic cell lines |
| ☒ | ☐ | Palaeontology and archaeology |
| ☐ | ☒ | Animals and other organisms |
| ☐ | ☐ | Clinical data |
| ☒ | ☐ | Dual use research of concern |
| ☒ | ☐ | Plants |

## Methods

| n/a | Involved in the study | |
|---|---|---|
| ☐ | ☐ | ChIP-seq |
| ☐ | ☐ | Flow cytometry |
| ☐ | ☐ | MRI-based neuroimaging |

## Eukaryotic cell lines

Policy information about cell lines and Sex and Gender in Research

| | |
|---|---|
| Cell line source(s) | Commercially available cell lines were used in this study available from ATCC. This is A172 cell line ( CRL-1620); M059J cell line (CRL-1620) and LN229 cell line (CRL-2611). |
| Authentication | We authenticated all cell lines used in this study based on the coverage of SNPs (ATAC-seq) compared to publicly available data of the same cell lines using NGScheckmate (https://github.com/parklab/NGSCheckMate). |
| Mycoplasma contamination | Cell cultures used for experiments providing data to this study were tested for myoplasm contamination and were found to be negative. |
| Commonly misidentified lines (See ICLAC register) | We used A172, as a model system for human glioblastoma as an example of mesenchymal like state. We validated the identity of this cell line using NGScheckmate. |

## Animals and other research organisms

Policy information about studies involving animals; ARRIVE guidelines recommended for reporting animal research, and Sex and Gender in Research

| | |
|---|---|
| Laboratory animals | We used Zebrafish (Danio Rerio) of the AB strain. Both male and female animals were used at age 48 hours post fertilization. |
| Wild animals | The study did not use wild animals. |
| Reporting on sex | Sex based analyses were not performed. Sex is not relevant for this study as we report an analysis software as main finding, therefore sex was not considered in the study design. |
| Field-collected samples | the study did not use field-collected samples. |
| Ethics oversight | All animal experiments were conducted according to the KU Leuven ethical guidelines and approved by the KU Leuven Ethical Committee for Animal Experimentation (approved protocol numbers ECD 000). |

Note that full information on the approval of the study protocol must also be provided in the manuscript.

# Clinical data

Policy information about clinical studies

All manuscripts should comply with the ICMJE guidelines for publication of clinical research and a completed CONSORT checklist must be included with all submissions.

Clinical trial registration | *Provide the trial registration number from ClinicalTrials.gov or an equivalent agency.*

Study protocol | *Note where the full trial protocol can be accessed OR if not available, explain why.*

Data collection | *Describe the settings and locales of data collection, noting the time periods of recruitment and data collection.*

Outcomes | *Describe how you pre-defined primary and secondary outcome measures and how you assessed these measures.*

# Plants

Seed stocks | *Report on the source of all seed stocks or other plant material used. If applicable, state the seed stock centre and catalogue number. If plant specimens were collected from the field, describe the collection location, date and sampling procedures.*

Novel plant genotypes | *Describe the methods by which all novel plant genotypes were produced. This includes those generated by transgenic approaches, gene editing, chemical/radiation-based mutagenesis and hybridization. For transgenic lines, describe the transformation method, the number of independent lines analyzed and the generation upon which experiments were performed. For gene-edited lines, describe the editor used, the endogenous sequence targeted for editing, the targeting guide RNA sequence (if applicable) and how the editor was applied.*

Authentication | *Describe any authentication procedures for each seed stock used or novel genotype generated. Describe any experiments used to assess the effect of a mutation and, where applicable, how potential secondary effects (e.g. second site T-DNA insertions, mosiacism, off-target gene editing) were examined.*

# ChIP-seq

## Data deposition

☐ Confirm that both raw and final processed data have been deposited in a public database such as GEO.

☐ Confirm that you have deposited or provided access to graph files (e.g. BED files) for the called peaks.

Data access links
*May remain private before publication.* | *For "Initial submission" or "Revised version" documents, provide reviewer access links. For your "Final submission" document, provide a link to the deposited data.*

Files in database submission | *Provide a list of all files available in the database submission.*

Genome browser session
(e.g. UCSC) | *Provide a link to an anonymized genome browser session for "Initial submission" and "Revised version" documents only, to enable peer review. Write "no longer applicable" for "Final submission" documents.*

## Methodology

Replicates | *Describe the experimental replicates, specifying number, type and replicate agreement.*

Sequencing depth | *Describe the sequencing depth for each experiment, providing the total number of reads, uniquely mapped reads, length of reads and whether they were paired- or single-end.*

Antibodies | *Describe the antibodies used for the ChIP-seq experiments; as applicable, provide supplier name, catalog number, clone name, and lot number.*

Peak calling parameters | *Specify the command line program and parameters used for read mapping and peak calling, including the ChIP, control and index files used.*

Data quality | *Describe the methods used to ensure data quality in full detail, including how many peaks are at FDR 5% and above 5-fold enrichment.*

Software | *Describe the software used to collect and analyze the ChIP-seq data. For custom code that has been deposited into a community repository, provide accession details.*

# Flow Cytometry

## Plots

Confirm that:

- [ ] The axis labels state the marker and fluorochrome used (e.g. CD4-FITC).
- [ ] The axis scales are clearly visible. Include numbers along axes only for bottom left plot of group (a 'group' is an analysis of identical markers).
- [ ] All plots are contour plots with outliers or pseudocolor plots.
- [ ] A numerical value for number of cells or percentage (with statistics) is provided.

## Methodology

| | |
|---|---|
| Sample preparation | *Describe the sample preparation, detailing the biological source of the cells and any tissue processing steps used.* |
| Instrument | *Identify the instrument used for data collection, specifying make and model number.* |
| Software | *Describe the software used to collect and analyze the flow cytometry data. For custom code that has been deposited into a community repository, provide accession details.* |
| Cell population abundance | *Describe the abundance of the relevant cell populations within post-sort fractions, providing details on the purity of the samples and how it was determined.* |
| Gating strategy | *Describe the gating strategy used for all relevant experiments, specifying the preliminary FSC/SSC gates of the starting cell population, indicating where boundaries between "positive" and "negative" staining cell populations are defined.* |

- [ ] Tick this box to confirm that a figure exemplifying the gating strategy is provided in the Supplementary Information.

# Magnetic resonance imaging

## Experimental design

| | |
|---|---|
| Design type | *Indicate task or resting state; event-related or block design.* |
| Design specifications | *Specify the number of blocks, trials or experimental units per session and/or subject, and specify the length of each trial or block (if trials are blocked) and interval between trials.* |
| Behavioral performance measures | *State number and/or type of variables recorded (e.g. correct button press, response time) and what statistics were used to establish that the subjects were performing the task as expected (e.g. mean, range, and/or standard deviation across subjects).* |

## Acquisition

| | |
|---|---|
| Imaging type(s) | *Specify: functional, structural, diffusion, perfusion.* |
| Field strength | *Specify in Tesla* |
| Sequence & imaging parameters | *Specify the pulse sequence type (gradient echo, spin echo, etc.), imaging type (EPI, spiral, etc.), field of view, matrix size, slice thickness, orientation and TE/TR/flip angle.* |
| Area of acquisition | *State whether a whole brain scan was used OR define the area of acquisition, describing how the region was determined.* |

Diffusion MRI ☐ Used ☐ Not used

## Preprocessing

| | |
|---|---|
| Preprocessing software | *Provide detail on software version and revision number and on specific parameters (model/functions, brain extraction, segmentation, smoothing kernel size, etc.).* |
| Normalization | *If data were normalized/standardized, describe the approach(es): specify linear or non-linear and define image types used for transformation OR indicate that data were not normalized and explain rationale for lack of normalization.* |
| Normalization template | *Describe the template used for normalization/transformation, specifying subject space or group standardized space (e.g. original Talairach, MNI305, ICBM152) OR indicate that the data were not normalized.* |
| Noise and artifact removal | *Describe your procedure(s) for artifact and structured noise removal, specifying motion parameters, tissue signals and physiological signals (heart rate, respiration).* |

Volume censoring | *Define your software and/or method and criteria for volume censoring, and state the extent of such censoring.*

## Statistical modeling & inference

Model type and settings | *Specify type (mass univariate, multivariate, RSA, predictive, etc.) and describe essential details of the model at the first and second levels (e.g. fixed, random or mixed effects; drift or auto-correlation).*

Effect(s) tested | *Define precise effect in terms of the task or stimulus conditions instead of psychological concepts and indicate whether ANOVA or factorial designs were used.*

Specify type of analysis: ☐ Whole brain ☐ ROI-based ☐ Both

Statistic type for inference | *Specify voxel-wise or cluster-wise and report all relevant parameters for cluster-wise methods.*

(See Eklund et al. 2016)

Correction | *Describe the type of correction and how it is obtained for multiple comparisons (e.g. FWE, FDR, permutation or Monte Carlo).*

## Models & analysis

| n/a | Involved in the study |
|---|---|
| ☐ | ☐ Functional and/or effective connectivity |
| ☐ | ☐ Graph analysis |
| ☐ | ☐ Multivariate modeling or predictive analysis |

Functional and/or effective connectivity | *Report the measures of dependence used and the model details (e.g. Pearson correlation, partial correlation, mutual information).*

Graph analysis | *Report the dependent variable and connectivity measure, specifying weighted graph or binarized graph, subject- or group-level, and the global and/or node summaries used (e.g. clustering coefficient, efficiency, etc.).*

Multivariate modeling and predictive analysis | *Specify independent variables, features extraction and dimension reduction, model, training and evaluation metrics.*
